# X-ray computed tomography images and network data of sands under compression

**DOI:** 10.1016/j.dib.2021.107122

**Published:** 2021-05-12

**Authors:** Wenbin Fei, Guillermo Narsilio, Joost van der Linden, Mahdi Disfani, Xiuxiu Miao, Baohua Yang, Tabassom Afshar

**Affiliations:** a Department of Infrastructure Engineering, The University of Melbourne, Parkville, Australia; b State Key Laboratory for Geomechanics and Deep Underground Engineering, China University of Mining and Technology, Xuzhou, Jiangsu Province 221116, China; c Information Science and Engineering School, Hunan Women's University, Changsha, Hunan Province 10004, China; d FSG Geotechnics and Foundations, Abbotsford, Australia

**Keywords:** Sand, X-ray CT, Network, Graph theory, Complex network model, Granular materials, Microstructure, Soil fabric

## Abstract

Ottawa sand and Angular sand consist of particles with distinct shapes. The x-ray computed tomography (XCT) image stacks of their in-situ confined compressive testings are provided in this paper. For each image stack, a contact network, a thermal network and a network feature - *edge betweenness centrality* - of each edge in the networks are also provided. The readers can use the image data to construct digital sands with applications of (1) extracting microstructural parameters such as particle size, particle shape, coordination number and more network features; (2) analysing mechanical behaviour and transport processes such as fluid flow, heat transfer and electrical conduction using either traditional simulation tools such as finite element method and discrete element method or newly network models which could be built based on the network files available here.

**Specifications Table**

**Table:** 

Subject	Geotechnical Engineering and Engineering Geology
Specific subject area	Microstructure characterisation in granular materials; Multiple scale analysis of thermal, hydraulic and geo-mechanical processes.
Type of data	XCT Image Network data file Network feature file
How data were acquired	XCT scanner Image analysis
Data format	Raw and analysed
Parameters for data collection	The pixel size of the XCT images is 13 µm. Four stages of axial stress applied to each sand specimen from 0 to 2.0, 6.1, 10.2 MPa.
	Sand particles were air-pluivated into an aluminium cylindrical container of a 25 mm diameter and 25 mm height. Each stage of axial stress was applied to the specimen and then allocated at Australian Synchrotron Imaging and Medical Beam Line (IMBL) to achieve sequential XCT images. Selected cubic sub-samples with a side length of 4.5 mm were cropped and attached to this paper. The images were post-processed using Otsu threshold segmentation, watershed segmentation and in-house code [1], [2] to construct contact and thermal networks. Based on the networks, edge betweenness centrality was calculated using a python library network [3], [4].
Data source location	Melbourne, Australia
Data accessibility	**Repository name:** Mendeley Data Data identification number: http://dx.doi.org/10.17632/szn4jtfkbx.1 **Direct URL to data:**https://data.mendeley.com/datasets/szn4jtfkbx/1 **Supplemnetary files to this paper**
Related research article	W. Fei, G.A. Narsilio, J.H. van der Linden, M.M. Disfani, Quantifying the impact of rigid interparticle structures on heat transfer in granular materials using networks, International Journal of Heat and Mass Transfer, 143 (2019) 118,514 [1]. W. Fei, G.A. Narsilio, Network analysis of heat transfer in sands, Computers and Geotechnics, 127 (2020) 103,773 [3]. J.H. van der Linden, G.A. Narsilio, A. Tordesillas, Thermal conductance network model for computerised tomography images of real dry geomaterials, Computers and Geotechnics, (2021) 104093. https://doi.org/10.1016/j.compgeo.2021.104093[5]

## Value of the Data

• Different particle shapes in Ottawa sand and Angular sand enable researchers to study the effect of particle shape on physical properties such as material stiffness, permeability, thermal conductivity [6].
• The XCT images can be considered as digital sand samples to conduct numerical experiments [6].
• The XCT images and the network files allow researchers to quantify sand microstructure at multiple length scales [3], [7], [8].
• Network features – *edge betweenness centralit*y – is provided as a benchmark.

## 1 Data Description

**Fig. 1:**
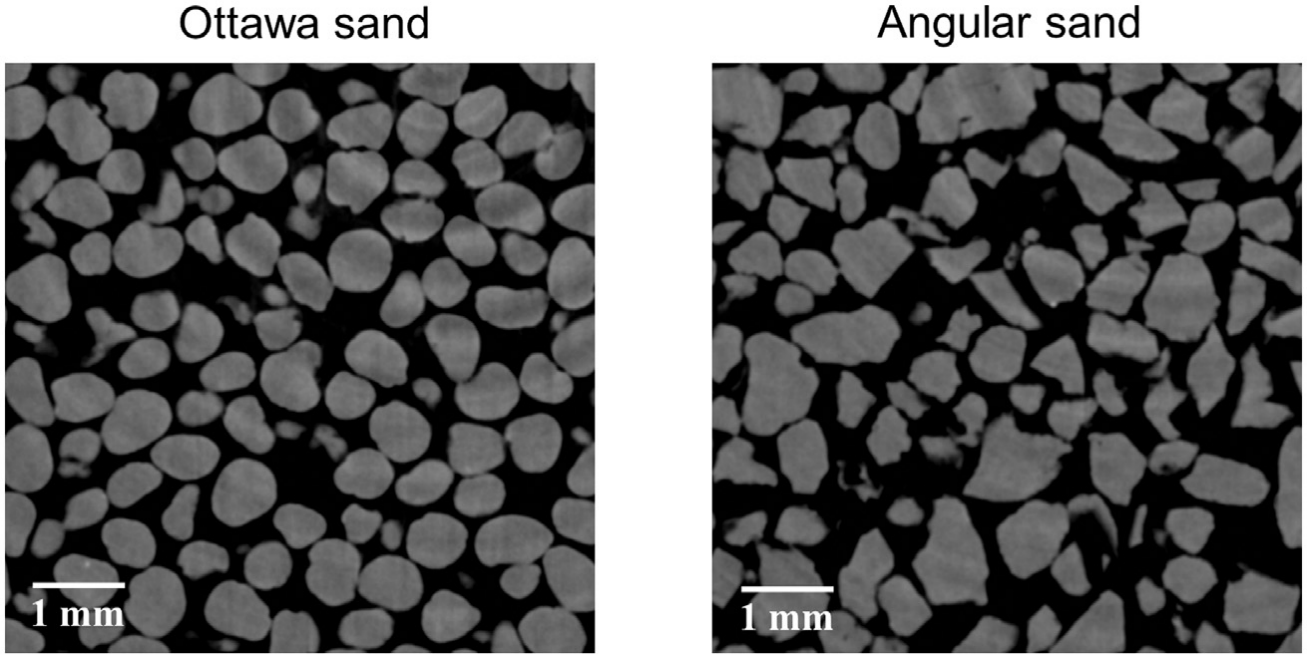
XCT scanned images of Ottawa sand and Angular sand.

Three levels of data are included in this paper. The first level is raw XCT images of Ottawa sand and Angular sand under four-stage axial compression and zero-lateral strain. The particle size and applied axial stress are summarised in Table 1. Each sand at every stress state was scanned, so eight XCT image stack are provided. Two slices from XCT images of Ottawa sand and Angular sand at rest are illustrated in Fig. 1.

**Table 1:** Particle size and axial compression stresses applied to each sample.

**Sand**	**Particle size (mm)**^a^	**Particle size (mm)**^b^	**Equivalent D_50_ (mm)**^b^	**Axial Stress (MPa)**
Ottawa sand	0.60–0.85	0.58–0.94	0.76	0, 2.0, 6.1, 10.2
Angular sand	0.60–1.18	0.39–0.99	0.68	0, 2.0, 6.1, 10.2

a Particle size from sieve analysis.

b Particle size calculated based on CT reconstructed sample.

**Fig. 2:**
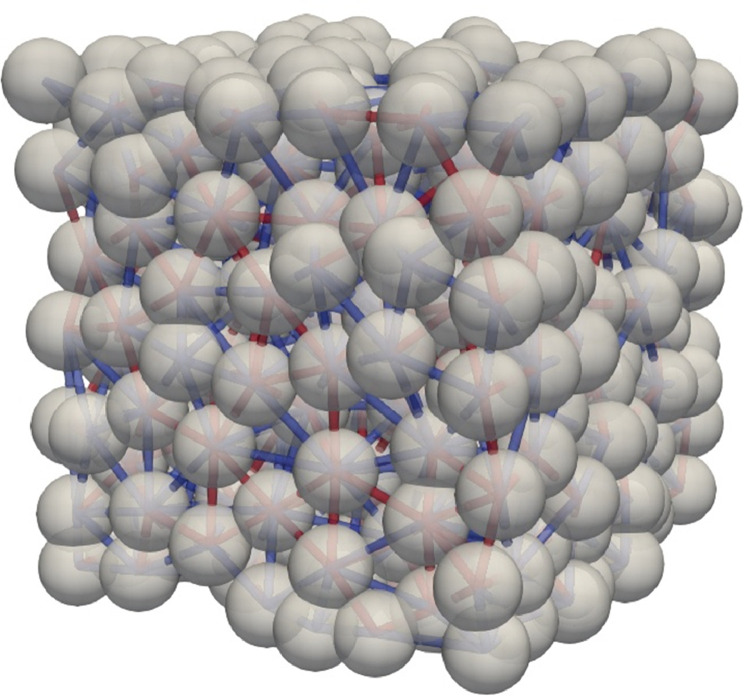
Thermal network of Ottawa sand at rest. Red edges represent interparticle contacts while blue edges represent near-contacts.

**Fig. 3:**
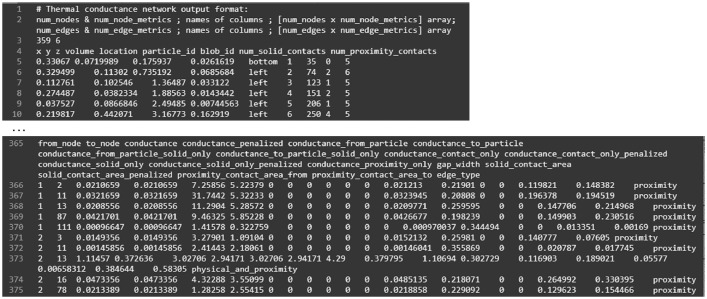
Format of a thermal network file, node section on the top while edge section on the bottom. Bolb_id is the identifier of each particle created during watershed segmentation. The meaning of the headings related to the network construction as explained in [1].

The second level provides contact network and thermal network files corresponding to each XCT image stack. A network is a web made of nodes and edges. In a contact network, a node is assigned to the centroid of each particle and an edge links two nodes representing two contacted particles. While nodes are the same in the thermal network (Fig. 2) of the same specimen, additional edges related to small gaps related to particle-air/fluid-particle heat transfer paths are generated. The small gaps are called near-contacts [1], [5]. In a thermal network file as shown in Fig. 3, it comprises of two sections related to nodes and edges. The edge type ‘proximity’ means that the edge is related to a near-contact while ‘physical_and_proximity’ indicates an interparticle contact. The reader only needs the coordinates of nodes (the first three columns in the node section) and the connection between nodes (the first two columns in the edge section). The meaning of other columns is related to the network construction which is detailed in [1].

**Fig. 4:**
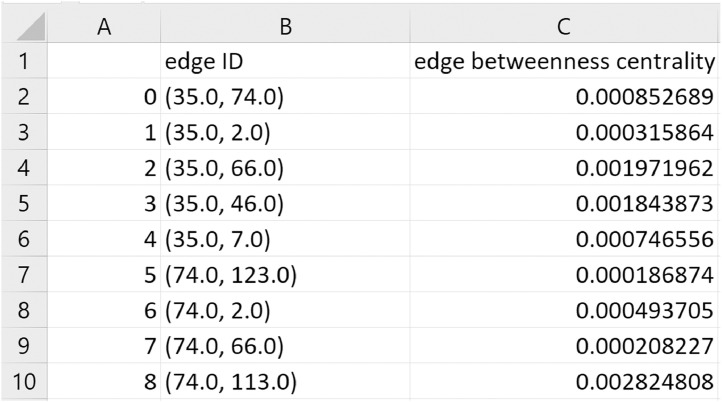
Format of network feature file.

Based on the networks, new microstructural can be computed using graph theory (i.e. complex network theory) [9], [10], [11]. A network feature – *edge betweenness centrality* – related to each edge is offered and its format is shown in Fig. 4. The edge ID is a combination of the ‘blob_id’ of two linked nodes from the network file.

## 2 Experimental Design, Materials and Methods

**Fig. 5:**
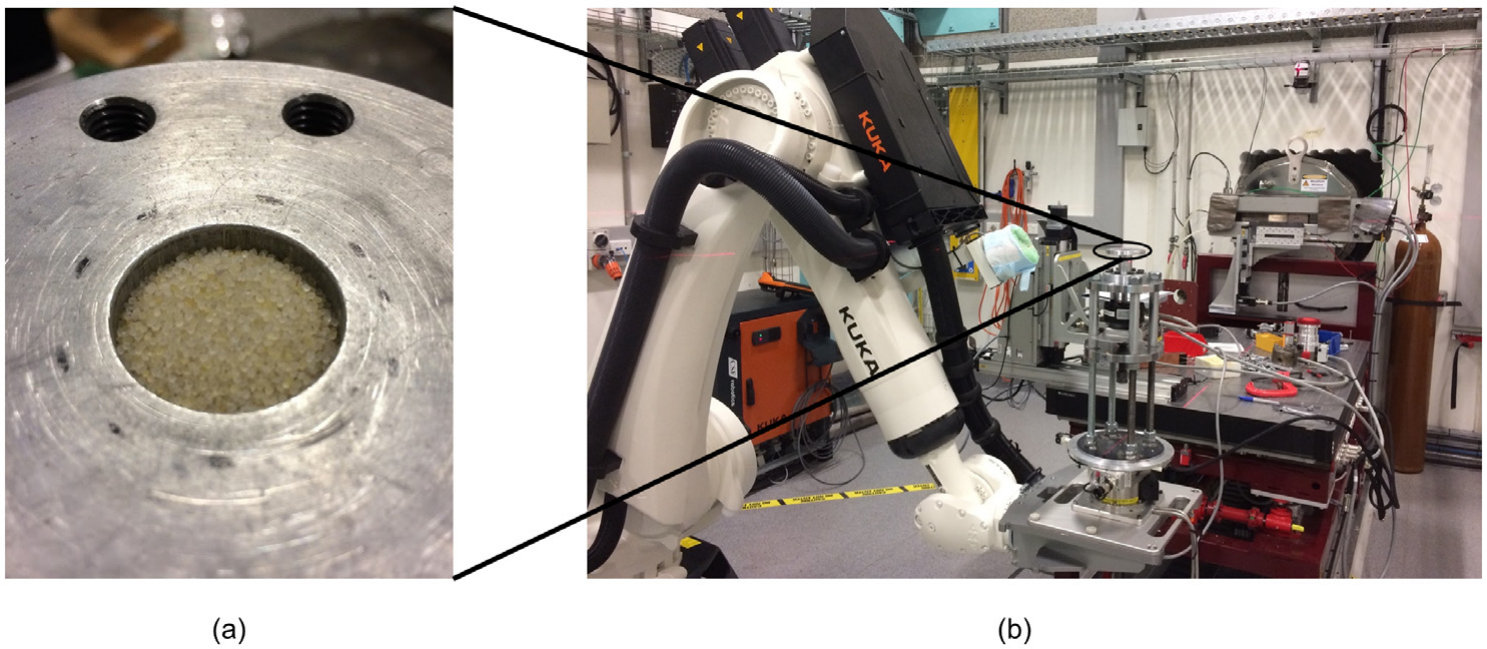
XCT workstation (a) and Ottawa sand in the sample container (b).

**Fig. 6:**
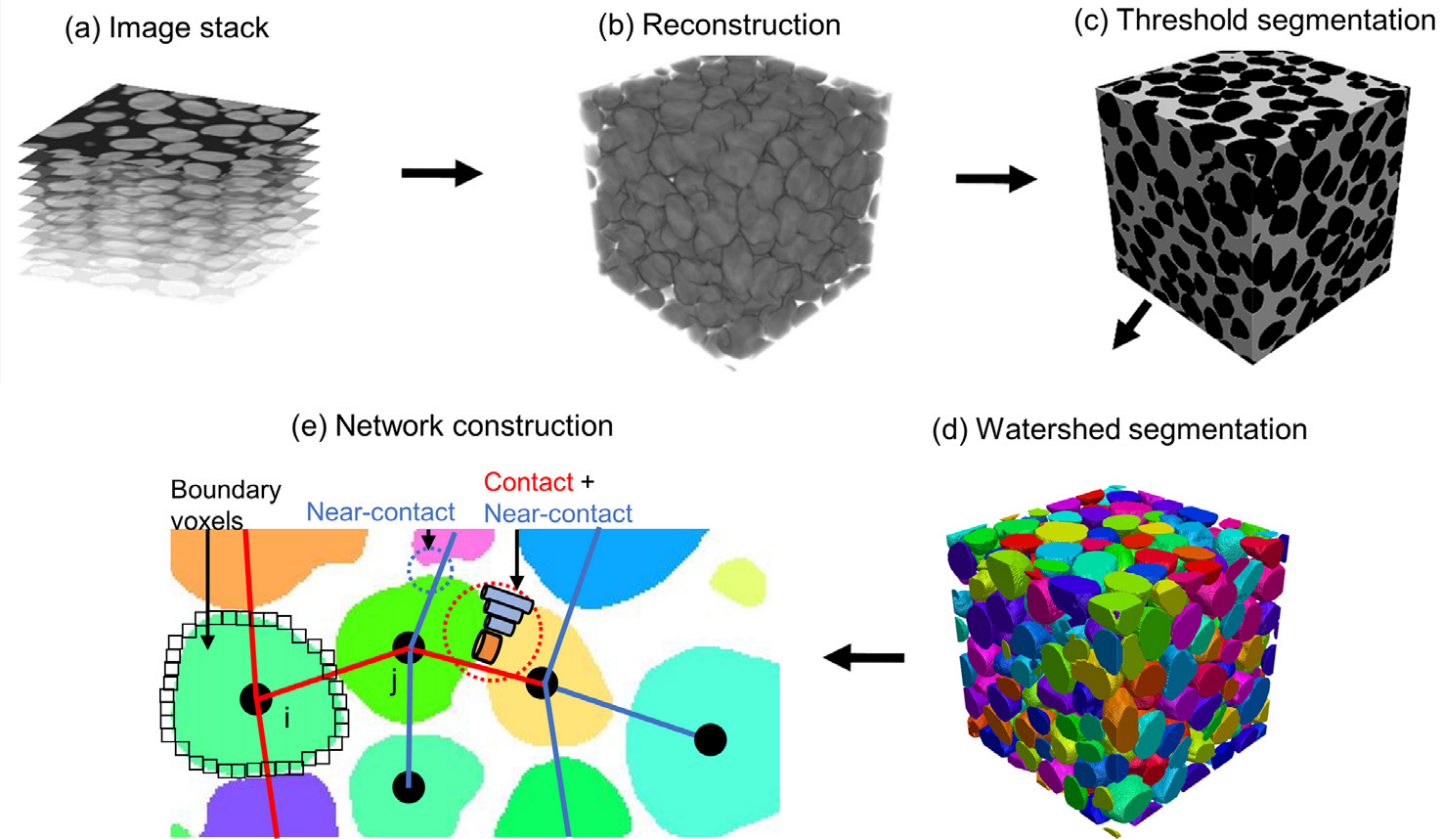
Procedures to construct a thermal network of Ottawa sand modified from [1].

Both Ottawa sand and Angular sand are made of silica. The two sands have a similar equivalent average particle size (Table 1) but different particle shape (Fig. 1). Each of them was air-pluviated in an aluminium container (Fig. 5(a)) which is connected to a loading frame on an XCT scanning platform (Fig. 5(a)). Each stress stage was loaded followed by penetrating the specimen using an X-ray with radiation energy of 60 keV to achieve sequential images as shown in Fig. 6(a). By stacking up the sequential image slices with one-pixel size (13 µm) spacing, a twin digital soil sample of the scanned sand was reconstructed as shown in Fig. 6(b). Next, Otsu threshold segmentation [12] was used to separate solid (black in Fig. 6(c)) and void phases (white in Fig. 6(c)). Watershed segmentation in MorphoLibJ [13] with a 6-voxel neighbourhood [14] was applied on the binary digital sample to achieve individual particles which were given unique IDs and rendered in random colour as shown in Fig. 6(d). For each particle, its boundary voxels were identified so the location of the particle centroid was computed by averaging the coordinates of the boundary voxels as shown in Fig. 6(e). If the boundary voxels of particle *i* is shared with its neighbouring particle *j*, an interparticle contact was detected and a corresponding network edge in red in Fig. 6(e) was generated. If the a small gap between two neighbouring particles and the distance between their boundary voxels was smaller than half of the average radius of all particles in the sample, an edge in blue in Fig. 6(e) representing near-contact was created in the thermal network. Removing the edges representing near-contacts from a thermal network is the contact network for the same specimen. By now, all edges in a network has the same contribution to the network and this type of network is call unweighted network. For a specific application, edges can be weighted by certain attribute to build weighted network [2], [3], [15]. For example, edges in a thermal network can be further weighted by thermal conductance to study heat transfer [3] and the method of computing thermal conductance was detailed in the paper [1].

## CRediT Author Statement

**Wenbin Fei:** Conceptualisation, Methodology, Software, Visualization, Writing–Original draft preparation; **Guillermo Narsilio:** Supervision, Investigation, Writing–Reviewing and Editing; **Joost van der Linden:** Software, Investigation; **Mahdi Disfani:** Investigation; **Xiuxiu Miao:** Investigation; **Baohua Yang:** Investigation; **Tabassom Afshar:** Investigation.

## Declaration of Competing Interest

The authors declare that they have no known competing financial interests or personal relationships which have or could be perceived to have influenced the work reported in this article.

## Appendix Supplementary materials

Image, application 1
