# Supplementary figures and images for: X-ray computed tomography images and network data of sands under compression
Source: Data Brief. 2021 May 12;36:107122. doi: 10.1016/j.dib.2021.107122 (PMC8165412; doi:10.1016/j.dib.2021.107122)

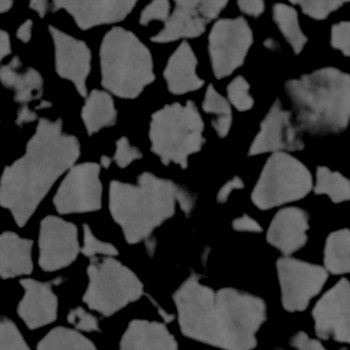

Supplement: Supplementary file 1 [file mmc1.zip › data/Angular-sand_0MPa/CT-image/original_greyscale.tif]

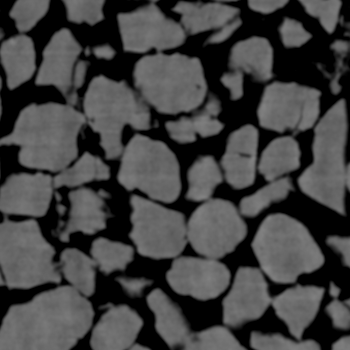

Supplement: Supplementary file 1 [file mmc1.zip › data/Angular-sand_10.2MPa/CT-image/original_greyscale.tif]

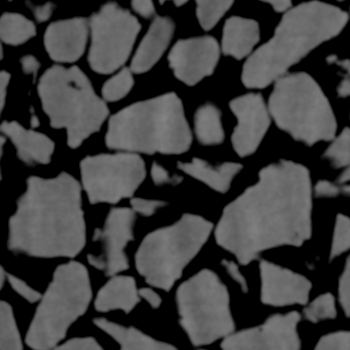

Supplement: Supplementary file 1 [file mmc1.zip › data/Angular-sand_2MPa/CT-image/original_greyscale.tif]

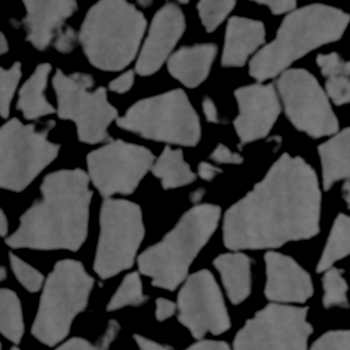

Supplement: Supplementary file 1 [file mmc1.zip › data/Angular-sand_6.1MPa/CT-image/original_greyscale.tif]

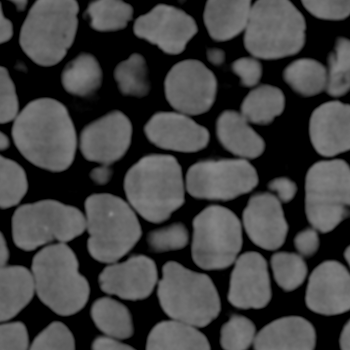

Supplement: Supplementary file 1 [file mmc1.zip › data/Ottawa-sand_0MPa/CT-image/original_greyscale.tif]

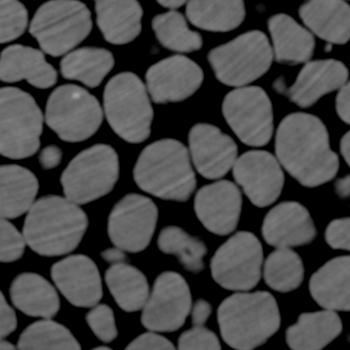

Supplement: Supplementary file 1 [file mmc1.zip › data/Ottawa-sand_10.2MPa/CT-image/original_greyscale.tif]

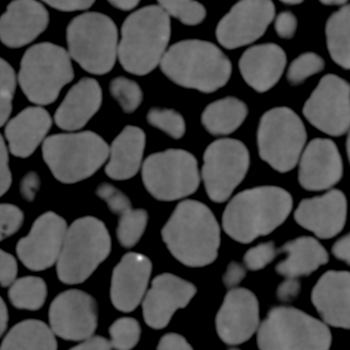

Supplement: Supplementary file 1 [file mmc1.zip › data/Ottawa-sand_2MPa/CT-image/original_greyscale.tif]

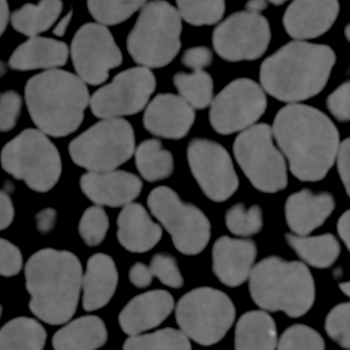

Supplement: Supplementary file 1 [file mmc1.zip › data/Ottawa-sand_6.1MPa/CT-image/original_greyscale.tif]
